# Effect of Alternative Protein Feeds on the Content of Selected Endogenous Bioactive and Flavour-Related Compounds in Chicken Breast Meat

**DOI:** 10.3390/foods9040392

**Published:** 2020-03-28

**Authors:** Vasiliki Gkarane, Marco Ciulu, Brianne Altmann, Daniel Mörlein

**Affiliations:** Department of Animal Sciences, University of Göttingen, 37075 Göttingen, Germany; vasiliki.gkarane@uni-goettingen.de (V.G.); brianne.altmann@agr.uni-goettingen.de (B.A.); daniel.moerlein@uni-goettingen.de (D.M.)

**Keywords:** *Arthrospira platensis*, spirulina, *Hermetia illucens*, black soldier fly, bioactive peptides, inosine, inosine-5´-monophosphate

## Abstract

Currently, soybean meal constitutes the main protein source for poultry production. However, the environmental and social issues related to soybean production are calling for more sustainable protein sources that can offset soybean requirements in animal production. *Hermetia illucens* larvae and the microalga spirulina (*Arthrospira platensis*) have proven to be effective alternatives to soybean meal for poultry production. In this study, the effect of 100% replacement of soy with partially defatted *Hermetia illucens* larvae and spirulina on the contents of selected endogenous bioactive (anserine, creatine and carnosine) and flavour-related (inosine and inosine-5´-monophosphate, IMP) compounds in chicken breast meat was evaluated. The results showed that the spirulina-based diet lowered the levels of anserine, carnosine and creatine compared to the control diet (3.3 vs. 4.1 mg/g, 0.15 vs. 0.72 mg/g and 1.49 vs. 2.49 mg/g, respectively) while IMP levels tended to be higher in spirulina-fed samples. Compared to the control group, *Hermetia illucens*-fed samples showed a lower content of bioactive peptides (anserine: 3.6 vs. 4.1 mg/g; carnosine: 0.39 vs. 0.72 mg/g; creatine: 2.03 vs. 2.49 mg/g), albeit to a lesser extent than the spirulina treatment group.

## 1. Introduction

Soybean meal (SBM) is included in the majority of poultry diets as the primary source of protein. Although the high nutritional value of soybean as a plant protein source guarantees efficient production, the social and environmental impact of the soybean industry has led to the need for more sustainable protein sources [1]. *Hermetia illucens* (black soldier fly, HI) larvae and the microalga spirulina (*Arthrospira platensis,* SP) have shown great potential as possible alternative protein feeds to be used in poultry production [2,3,4,5,6,7,8]. For example, the study by Neumann et al. reveals how a broiler diet with 100% replacement of soybean meal by HI larvae with a proper amino acid supplementation can result in a superior growth, feed conversion ratio and protein deposition [9]. On the other hand, a dietary substitution with SP and amino acid supplementation only resulted in comparable body weights and a feed conversion ratio similar to that of the SBM control group. Although great strides in understanding the effects of larvae and microalgae have emerged from an animal nutrition and physiology standpoint, to the best of our knowledge, the scientific literature still offers scarce information regarding the content of bioactive substances in meat products derived from the feeding of these biomasses.

Peptides like carnosine, anserine and creatine are predominant bioactive compounds found in poultry meat and are associated with many physiological functions and health benefits (i.e., anti-glycation, antioxidant and antiaging properties, as well as pH-buffers in muscle) [10,11,12,13,14,15]. In animal muscle, carnosine is synthesised from histidine (His) and β-alanine (β-Ala) through carnosine synthase, whereas anserine is a methylated analogue of carnosine [16]. Several studies have reported the effect of dietary supplementation of His or β-Ala on peptide concentration in chicken muscle [17,18,19,20], since the depletion of these peptides can negatively impact meat quality and nutritional value [17]. On the other hand, creatine is synthesised from L-arginine, glycine and L-methionine; its role is to maintain adenosine triphosphate (ATP) concentrations so as to supply energy to muscles [10]. Inosine 5´-monophosphate (IMP) is the major nucleotide in muscle [21]. It is formed during rigor mortis from ATP, and affects the meat’s flavour by contributing to the umami taste [22]. In addition, the concentration of IMP and inosine, (i.e., its corresponding nucleoside), has been associated with a higher taste intensity in chicken breast [23].

Since the intake of these mentioned compounds is either beneficial to human health or affects the sensory properties of chicken meat, it is important to identify ways to assess, yet also retain, their concentration in meat. Accordingly, the levels of carnosine, anserine and creatine along with the IMP content have been previously taken into account to assess the quality of chicken meat [16].

In the present study, we evaluated the content of the selected compounds in chicken breast originating from previous experimental trials [9], which aimed at investigating the effect of 100% soybean meal substitution with SP and HI on meat quality. 

## 2. Materials and Methods 

### 2.1. Birds and Diets

The study was carried out at the Department of Animal Sciences, University of Goettingen, and was approved by the Ethics Committee of the Lower Saxony Office for Consumer Protection and Food Safety (LAVES), Germany [9]. The experiment was divided into a starter feeding phase (1–21 d) and a grower feeding phase (22–34 d). One-day-old male chickens (Ross 308) were randomly allocated to floor pens (six pens per treatment group; seven animals per pen), with 5.8 birds / m^2^. Average body weights per pen were similar at the start of the study. Feed and water were available ad libitum. The control diet (C) was based on wheat, corn and SBM as the main ingredients. The experimental diets replaced SBM with either 100% partially defatted HI or 100% SP, while supplementing amino acids according to breeder guidelines. All surviving animals were slaughtered at 35 days of age at an average slaughter weight of 1.75 (± 0.24) kg. Out of the resulting 113 carcasses, 46 birds were randomly selected (16 SBM, 16 HI and 14 SP). Breast muscle (M. pectoralis major) samples were vacuum-packed in PA/PE bags and stored at −20 °C until analysis. Full details of the animal and production characteristics and composition of feeds are published in Neumann et al. [9] (Experiment 3).

### 2.2. Chemicals and Reagents

Analytical standards of inosine-5´-monophosphate (IMP), inosine, anserine, carnitine and creatine were purchased from Sigma-Aldrich (Munich, BY, Germany). Methanol (HPLC grade) and acetonitrile (HPLC grade) were obtained from VWR Chemicals (Darmstadt, Germany). Trichloroacetic acid (TCA) was purchased from Carl Roth® (Karlsruhe, BW, Germany). Ammonium acetate, KH_2_PO_4_ and tetrabuthylammonium hydrogen sulphate (TBAHS) were procured from Sigma-Aldrich (Munich, BY, Germany). Ultrapure water was used for all analyses.

### 2.3. Analysis of Peptides

The carnosine, anserine and creatine contents were determined using the method of Jung et al. [16] with some modifications. Minced samples (2.5 g) were homogenised with 1 ml of 0.01 N HCl (aq) at 1160 rpm (Schuett-homgen^plus^ homogenizer, Schuett-biotec GmbH, Germany) for one min. The extracts were centrifuged at 4 °C for 15 min at 14800 × g (Pico & Fresco 17/21 centrifuge, Thermo Electron LED GmbH, Osterode, NE, Germany). The supernatant (250 µL) was mixed with acetonitrile (750 µL) for one min, then rested at 4 °C for 20 min. Finally, the sample was centrifuged (14,800 × g) for 15 min at 4 °C. 

HPLC analyses were performed by means of an L-7100 pump (Merck Hitachi, Darmstadt, HE, Germany), an L-7200 autosampler (Merck Hitachi, Darmstadt, HE, Germany) and an L-4250 UV-Vis detector (Merck Hitachi, Darmstadt, HE, Germany) operating at 214 nm. A Merck SeQuantR ZIC-HILIC column (150 × 4.6 mm, 5 µm) was maintained at 20 °C in a 5310-column oven (VWR Hitachi Chromaster). Eluent A contained 0.65 mM ammonium acetate in a water/acetonitrile mix (25/75, v/v, pH 5.5) and eluent B contained 4.55 mM ammonium acetate in a water-acetonitrile mix (70/30, v/v, pH 5.5). The mobile phase was supplied at 1.4 mL/min for 16 min with a linear gradient (0%–100% B). Quantification was performed by the external calibration method. Identification of the analytes was performed by the comparison of the retention time and by spiking the peak with a standard solution containing known amounts of the analytes. All analyses were performed in duplicate. 

### 2.4. Analysis of IMP and Inosine

The IMP and inosine content was determined using the method by Morzel and Van De Vis (2003) with some modifications [24]. Minced samples (0.2 g) were homogenized with 1 ml of 5% (w/v) TCA (aq) for one min at 1600 rpm followed by chilling on ice for 15 min. The liquid extract was centrifuged at 4 °C for five min at 12,000 × g. The supernatant (200 µL) was diluted 1:4 with 5% (w/v) TCA (aq) at pH 7.0. Extracts were kept at −20 °C before being injected in the HPLC system. The system (VWR Hitachi, Chromaster) was equipped with a 5260 pump, a 5260 autosampler (injection volume: 10 µL), and a 5410 Diode Array Detector operated at 260 nm. A LiChroCart Licrosphere 100 RP8 (250 × 4.6 mm, 5 μm) column was maintained at 30 °C in a 5310-column oven. The mobile phase consisted of 100 mM KH_2_PO_4_ (aq), 1.44 mM TBAHS (aq) and 0.5% methanol (aq, pH 7.0). Quantification was performed by the external calibration method. Identification of the analytes was performed by the comparison of the retention time and by spiking the peak with a standard solution containing known amounts of the analytes. All analyses were performed in duplicate.

### 2.5. Statistical Analysis

Data were analysed with the GLM procedure in SAS Enterprise Guide (v9.4, SAS Institute Inc, Cary, NC, USA). A Levene´s test (*p* < 0.05) was performed to check the homogeneity of variances. A Box–Cox transformation was applied to non-normally distributed variables. A one-way analysis of variance (ANOVA) and a Tukey’s test were used to compare the significant differences between dietary groups (*p* < 0.05). 

## 3. Results

The peptide content was generally lower in the chickens fed with SP compared to the chickens fed with the other two diets. Specifically, SP chickens had a lower (*p* < 0.05) anserine content than chickens fed with the C diet; no significant differences (*p* > 0.05) were observed between the C and HI groups. Furthermore, levels of carnosine in the SP group were considerably lower (*p* < 0.001) than in the C group, with the former showing only 21% of the carnosine content of the latter. HI samples showed a lower content (*p* < 0.001) of carnosine than the C group, although to a lesser extent (54%). Finally, creatine showed a similar trend to carnosine, with SP samples exhibiting the lowest content (*p* < 0.001) followed by the HI and C group. Chickens fed with the SP diet had a higher (*p* < 0.05) IMP content than chickens fed with the HI diet, but were on par with the control (C) group. Inosine concentration did not show any significant difference among the three groups. All results are shown in Table 1. 

## 4. Discussion

### 4.1. Peptides

As reported in other studies [10,16], anserine was the most abundant dipeptide among the three compounds studied, followed by creatine and carnosine, regardless of the diet. 

Primarily, the factors that may affect the concentration of the selected dipeptides are the type of muscle fibre, species/genotype, animal gender or cooking method [10,16,23,25,26]. However, Juniper and Rymer [25] have reported similar contents of these peptides between different species (pheasant vs. free range chicken), implying that the dietary or environmental impact may be stronger than the impact of species. 

Similarly, in the current study the differences in amino acid composition of the dietary ingredients, i.e., synthetic supplements, may have affected the peptide content. Specifically, the SP diet had a lower histidine content than the other two diets (4.6 vs. 5.4 g/kg and 5.5 g/kg for the C and HI diet, respectively, for the starter period; and 3.9 g/kg diet vs. 4.9 and 5.0 g/kg for the C and HI diet, respectively, for the grower period [9]). This observation could explain the low amounts of carnosine observed for the SP samples, since histidine is one of its two main components.

Regarding the carnosine content of the HI samples, it is interesting to note that, although the HI meal had a histidine content comparable to that of the soybean meal, the levels of carnosine in HI meat were still lower than in the C group. This observation suggests that factors other than the content of histidine in the feed could play a role in the final carnosine levels in meat. For example, in previous studies [2,27] it has been observed that the apparent ileal digestability (AID) of histidine in black soldier fly larva meal for broilers is lower than the one observed for the other amino acids. On the other hand, in the case of soybean meal, the AID of histidine appeared to be comparable to that of the other protein sources [28]. Previous studies have also shown that the dietary levels of β-alanine influence the content of carnosine in muscle [18,27,28]. However, the absence of data regarding the β-alanine content in the three diets prevented us from making conclusions. Lower levels of histidine in the SP diet could also underlie the differences in the anserine content. Nonetheless, as shown by our data and previously reported elsewhere [17], the dependency on the dietary content of histidine is less considerable for anserine as compared to carnosine.

Creatine synthesis is enhanced by arginine and glycine supplementation in chicken meat, while methionine has a limited role [29]. On the one hand, levels of arginine and methionine in the three diets were comparable, but on the other hand, no information regarding the dietary glycine content was available in the present study. However, the expression of two of the enzymes involved in creatine biosynthesis in vertebrates (i.e., L-arginine: glycine amidinotransferase (AGAT) and guanidine acetate methyl-transferase (GAMT)) can be modulated by other dietary factors [30].

The beneficial effects of the ingestion of bioactive peptides on human health still need to be further investigated. Nevertheless, in the light of what has been observed, it is logical to assume that low contents of histidine-containing peptides could be associated with a reduced scavenging capacity and a lower protective effect against cardiovascular diseases [31]. In addition, lower levels of creatine may affect the potential therapeutic effect against neurodegenerative disorders [14] as well as the benefits on athletic performance [15].

### 4.2. IMP and Inosine

According to Wang et al. [32], the taste of chicken is influenced by IMP, which in turn is affected by dietary purine nucleotides. These compounds are enzymatically hydrolysed into pentose, phosphoric acid and purines before being transferred into tissues (liver, kidney, muscle) and resynthesised in the de novo synthesis of IMP. In their study, Wang et al. [32] reported a higher IMP content in broiler meat that was supplemented with purine nucleotides (compared to a control diet based on corn and soybean meal without supplementation). They also reported that diets supplemented with purine nucleotides inhibited the activity of 5´-nucleotidase (an enzyme that contributes to IMP degradation), thus enhancing IMP concentration. Kaneko et al. [33] classified SP as a supplement with a very high content of dietary purines (more than 1000 mg/100 g). Thus, the higher IMP content found in the SP group may be associated with the purine nucleotide concentration of this dietary treatment. 

## 5. Conclusions

To the best of our knowledge, this is the first study regarding the effect of alternative protein sources on endogenous bioactive and flavour-related compounds in chicken meat. The preliminary results suggest that, especially in the case of spirulina as a protein source, particular attention should be paid to the levels of amino acids (e.g., histidine) during feed formulation in order to maintain meat peptide levels comparable to that of the current product. Further research is needed in order to draw solid conclusions on the effects of 100% replacement of soybean meal with either spirulina or *Hermetia* larvae meal on bioactive and taste-related compounds. For instance, an in-depth nutrition study specifically dedicated to investigating the content of the selected compounds in the final product should be developed. Nonetheless, in the light of the present findings, breeders switching to alternative dietary sources may face conflicting objectives: they will be challenged to ensure a product of acceptable flavour and high nutritional value while retaining the optimal levels of these compounds.

## Figures and Tables

**Table 1:** IMP, inosine and peptide content (mg/g of breast meat) in chickens fed with *Hermetia illucens* (HI), spirulina (SP) and control diets (C).

				Diet			*p*-level
	Calibration Range (µg/mL)	Calibration Curve Equation	R^2^	C (*n* = 16)	SP (*n* = 14)	HI (*n* = 16)	
Anserine	50–300	y = 7 × 106x − 62,938	0.9993	4.14 ± 0.20 ^a^	3.29 ± 0.21 ^b^	3.64 ± 0.24 ^ab^	0.035
Carnosine	50–300	y = 1 × 107x − 58,900	0.9993	0.72 ± 0.06 ^a^	0.15 ± 0.02 ^c^	0.39 ± 0.03 ^b^	<0.001
Creatine	50–300	y = 6 × 106x + 29,776	0.9997	2.49 ± 0.09 ^a^	1.49 ± 0.11 ^c^	2.03 ± 0.11 ^b^	<0.001
IMP	20–600	y = 28,458x + 169,871	0.9999	1.18 ± 0.12 ^ab^	1.35 ± 0.14 ^a^	0.86 ± 0.08 ^b^	0.007
Inosine	2–80	y = 43,196x − 40,175	0.9997	1.55 ± 0.08	1.76 ± 0.09	1.54 ± 0.06	0.100

Content of the selected compounds is expressed as mean ± SEM. Values with no superscripts in common differ significantly (*p* < 0.05).
